# Investigating the Efficacy of *Tasmannia lanceolata* Extract in Inactivating Fungi and Prolonging the Shelf Life of Date Fruit

**DOI:** 10.3390/foods11172631

**Published:** 2022-08-30

**Authors:** Fahad Al-Asmari, Saleha Akter, Ram Mereddy, Yasmina Sultanbawa

**Affiliations:** 1 Department of Food Science and Nutrition, College of Agriculture and Food Sciences, King Faisal University, P.O. Box 400, Al-Hofuf 31982, Saudi Arabia; 2 ARC Industrial Transformation Training Centre for Uniquely Australian Foods, Centre for Nutrition and Food Sciences, Queensland Alliance for Agriculture and Food Innovation (QAAFI), The University of Queensland, Elkhorn Building (#1024), 80 Meiers Road, Indooroopilly, QLD 4068, Australia; 3 Department of Agriculture and Fisheries, Queensland Government, Health and Food Sciences Precinct, 39 Kessels Rd, Coopers Plains, QLD 4108, Australia

**Keywords:** *Tasmannia lanceolata*, Tasmanian pepper, polygodial, Date fruit, *Phoenix dactylifera*, shelf life, post-harvest, spoilage, fungi

## Abstract

Date palm (*Phoenix dactylifera* L.) is one of the world’s oldest cultivated plants. Post-harvest losses of date palm due to fungal contamination reached up to 50% of the total production. This study aimed to investigate the potential of the extract of Tasmanian pepper leaf (TPL) and the non-thermal treatment of photosensitization mediated by curcumin in reducing the fungal contamination and enhancing the shelf life of date palm. In the *in vivo* storage study, the dates were treated with three different concentrations of TPL extract 12.5, 25, and 50 µg/mL and stored at 30 °C. The findings obtained for the treatment with TPL extract exhibited potent antifungal activity against most of the tested fungi, where minimum inhibitory concentrations (MICs) and minimum fungicidal concentrations (MFCs) were < 25 µg/mL for polygodial, the bioactive compound in TPL. The shelf life of date palm treated by 50 µg/mL polygodial was extended up to 21 days, thrice as much as the untreated controls. In contrast, a lower concentration of TPL extract (25 µg/mL polygodial) revealed up to a 15-day shelf life extension compared to untreated dates (7 days). The results obtained from the study suggested that using TPL extracts against pathogenic and spoilage fungi occurring in fresh date fruits is a promising treatment for the shelf life extension of fresh date fruits at room temperature.

## 1. Introduction

Date fruits have recently received greater attention owing to their health benefits and economic values in many countries. Fresh date fruits have a short shelf life as it is highly perishable due to their high moisture, sugars, and alkaline pH (>8). This shelf life limitation could be the main challenge for farmers and marketers during transport, storage, and export. Microbial contamination of dates can happen at different post-harvest stages during improper handling, transportation, and under poor storage conditions with fluctuating temperatures [1]. The contamination in dates is due to a mixture of bacteria and fungi. This, in turn, plays an essential role in determining both safety and shelf life. Among these microorganisms, fungi are the leading cause of food-borne diseases and spoilage in dates, while the plant pathogenic fungus *Aspergillus niger* is the predominant species associated with dates [2]. The presence of spoilage fungi in dates could lead to fruit rot and quality deterioration [3]. Spoilage of dates caused by fungi is widespread, causing significant post-harvest losses, while the presence of mycotoxins in dates and their products has been reported [2,4,5,6]. The issue with the current preservative agents either relates to their insufficient ability to reduce or eliminate these microbes properly from the surface of dates or the emergence of antifungal-resistant strains. There is, therefore, a need to find alternative treatments as interventions to reduce post-harvest losses in fresh date fruits. Native plant extracts from various species grown throughout Australia, including *Tasmannia lanceolata,* have been proven to affect fungal growth *in vitro* and *in vivo* [7]. *T. lanceolata,* or Tasmanian pepper, possessed the strongest fungicidal activity among Australian native plant extracts [8]. Tasmanian pepper leaf (TPL) contains polygodial, the principal pungent and bioactive component [9]. Polygodial is a drimane-type sesquiterpene dialdehyde and has been reported to have the antimicrobial, antifungal, and antiparasitic potential [10]. Polygodial has shown potent antimicrobial properties against a broad spectrum of pathogenic microbes [7]. Several studies have reported the therapeutic properties of polygodial against filamentous molds and yeast-like fungi and yeasts [11,12,13,14,15,16]. Polygodial can enter the cell membrane of microbes via passive diffusion, and once inside, it may react with various intercellular structures causing cell dysfunction [17]. The antifungal mechanism of Polygodial is reported to be by acting preferentially on the primary amine groups of phosphatidylethanolamine and phosphatidylserine in the outer monolayer of the plasma membrane [17]. Its potency exhibited no sensitivity to conditions such as temperature and pH, whereas it increased when tested under acidic conditions [18]. Despite its fungicidal activity, until recently, there have been minimal rigorous studies referring to the uses of TPL extracts as a post-harvest intervention treatment. TPL extract is an emerging natural preservation technology that the industry could utilize to disinfect the surface of date fruit, thereby extending the shelf life of dates. 

Therefore, this current study was undertaken to determine the potential antifungal activity of the TPL extract *in vitro* against seven plant pathogenic and spoilage fungi associated with dates and *in vivo* dipping of fresh dates in TPL extract to assess the extension in storage life of fresh dates. The objectives of this study were, therefore, to evaluate the effect of TPL extract on fugal spore survival; to determine the minimum inhibitory concentration (MIC) and minimum fungicidal concentration (MFC) of each tested fungi; and to assess the TPL efficacy in extending the shelf life of fresh dates.

## 2. Materials and Methods

### 2.1. Preparation of Plant Extract

The water-soluble powder containing 10% Tasmanian pepper leaf (TPL) extract and 90% Maltodextrin was purchased from Essential oils of Tasmania Pty Ltd (Margate, Tasmani, Australia). The initial TPL stock mixture solution was prepared by dissolving 5 g of powder in 10 mL of sterile water. Then the principle bioactive compound, polygodial in the mixture solution, was determined using HPLC.

Each one gram of TPL initial stock mixture solution contained 5500 µg/mL polygodial at pH 4.6. Accordingly, eight typical dilutions; 50, 100, 200, 300, 400, 500, 600 and 700 µg/mL polygodial were then prepared. In the present study, water-soluble TPL extract powder has been chosen over non-water soluble extracts such as essential oils or oleoresins because non-water soluble extract requires incorporating an emulsifier or solvent into the test medium to ensure contact between the test microorganism and the agent during *in vitro* and *in vivo* experiments.

Maltodextrin, which forms 90% of TPL extract powder, is produced mainly from starch by partial hydrolysis and utilized mainly in food and pharmaceutical products. Although Maltodextrin was not tested alone against fungi, it is unlikely to possess antifungal properties.

### 2.2. Preparation of Fungal Spore Suspensions

Seven pathogens and food spoilage fungi were obtained from the American Type Culture Collection (ATCC) (Manassas, VA, USA). The fungi were *Aspergillus niger* ATCC 6275, *Aspergillus flavus* (ATCC 9643), *Penicillium griseofulvum* (ATCC 48927), *Penicillium chrysogenum* (ATCC 10106), *Fusarium oxysporum* (ATCC 62606), *Candida albicans* (ATCC 10231) and *Zygosaccharomyces bailii* (ATCC 42476). The aqueous suspensions of mold spores were obtained by resuscitating them on the potato dextrose agar (PDA) at 25 °C for 7 days. Following this, the spores were harvested by flooding the plate with 10–15 mL of phosphate buffer solution (PBS) containing 0.1% Tween 80 solution and rubbing the surface mycelium gently with a spreader to release the spores into an aqueous suspension. At the same time, the yeast was enriched in broth media at 30° for 48 h. The supernatant was decanted, and the pellet was re-suspended in PBS by vortexing. The inoculum size of the spores was adjusted between 10^3^ and 10^5^ spores/mL using peptone water. All fungal species used in this experiment were either similar to those isolated previously from fresh dates or have been reported as fungi associated with dates. Using ATCC instead of isolated species was due to the Australian Quarantine & Inspection (AQIS) that prohibits work on any imported fresh product unless otherwise permitted. The permitted products and isolated microorganisms can only be tested in a quarantine-approved laboratory. ATCC cultures were used to enable further analysis in other laboratories.

### 2.3. Disc Diffusion Assay

Aliquots of 10 μL of each TPL extract dilution (50, 100, 200, 300, 400, 500, 600, and 700 were pipetted on a sterile paper disc (Whatman No1, 6 mm paper disc) on the solid media. Three discs were placed in each Petri dish and then incubated at the optimal growth temperatures of 25 °C/5 days for mold and 30 °C/48 h. for yeast. Fungal inhibition was determined by measuring the diameter of the clear zone of inhibition of growth around each disc and recorded as the diameter of the inhibition zone in millimeters. All assays were performed in triplicate of each sample.

### 2.4. Serial/Micro Dilution Assay

To determine the minimum inhibitory concentrations (MICs) and minimum fungicidal concentrations (MFCs), prepared serial dilutions of TPL extract of 50, 100, 200, 300, 400, 500, 600, and 700 µg/mL polygodial were assessed on the suspended fungal spores as described by Schwalbe, Steele-Moore [19] with modifications. Briefly, an aliquot of 1 mL of each TPL extract dilution (≥pH 5.5) was mixed with the same amount of fungal suspension (1:1 *v*/*v*). The spores-TPL extract mixtures were then incubated at room temperature for periods of 4, 8, 12, 16, 20, and 24 h. Aliquots of 100 µL of the incubated mixture were transferred into PDA plates containing 100 mg/L chloramphenicol and then incubated at 25 °C/5 days for mold and 30 °C/48 h for yeast to confirm the viability of the spores. Grown colonies were counted and expressed as spores /mL.

All assays were performed in triplicate of each sample. The endpoint criterion of MIC was defined as the lowest concentration of TPL extract to induce no fungal growth after the incubation period. The endpoint criterion of MFC_90_ was defined as the lowest concentration of TPL extract inducing fewer than three colonies, which corresponded to killing approximately 90% of fungi after the incubation period.

### 2.5. Treatment of Date Fruits (In Vivo)

Fresh dates of *Barhi* cultivar at the *Khalal* stage were purchased from Gurra farm, South Australia, April 2016. The samples were received fresh, yellow in color, and crunchy in texture and immediately kept at −20 °C and used within 3 months. Three dilutions of TPL extract containing 12.5, 25, and 50 µg/mL, pH 5.7, were applied to the date fruits. In brief, 30 date fruits without damage or disease symptoms were selected and washed gently using running water. The fruits were then dipped into three different TPL dilutions for different periods of soaking (0, 20, and 40 min), followed by air-drying for about 15 min. The fruits were then packed in sterile plastic bags and stored at 30 °C. The shelf life of treated dates was assessed visually based on fungal growth present on the skin of the date, as described in Table 1. Thus, any fungal growth (slight, moderate, or severe) observed on the outer skin of date fruit was considered the endpoint of the date’s shelf life.

### 2.6. Statistical Analysis

Statistical analysis was performed using the XLSTAT-Pro software package version 7.0 (XLSTAT Addinsoft, Paris, France). One-way analysis of variance (ANOVA) and Tukey HSD Tests were used to determine statistical differences. Means were considered to be significantly different at *p* < 0.05.

## 3. Results and Discussion

### 3.1. Antifungal Activity of TPL Extract (In Vitro)

**Figure 1:**
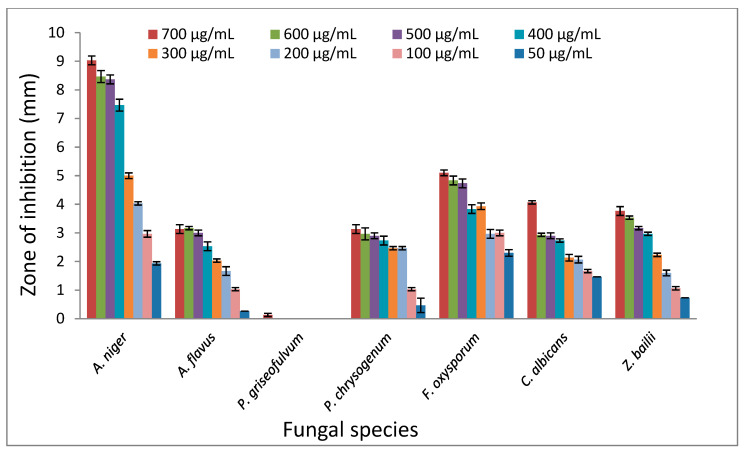
The effect of different concentrations of Tasmanian pepper leaf (TPL) extract on the fungal species’ inhibition zones.

The antifungal activity of TPL extracts serial dilutions were performed using agar disk and serial dilution assays against the spores of seven fungal species. Disk diffusion assay revealed inhibiting zones with significant variation (*P* < 0.05) against all fungal species, except *P. griseofulvum*, which did not show any appreciable activity. The mean diameters of the clear zones of TPL extract against tested fungal spores are shown in Figure 1.

TPL extract ranged from 50–700 μg/mL, showing a higher overall inhibition (2–9 mm) against *A. niger* followed by *F. oxysporum* (2.3–5.1 mm), *C. albicans* (1.46–4 mm), *Z. bailii* (0.73–3.76 mm), *P. chrysogenum* (0.46–3.1 mm) and *A. flavus* (0.26–3.1 mm) under the same conditions. Interestingly, the *F. oxysporum* showed an almost constant diameter zone of inhibition over all tested TPL extract dilutions, which may indicate its high susceptibility to TPL extract. Conversely, TPL extract showed poor potency against *P. griseofulvum* spores with a < 0.1 mm zone of inhibition. 

The result of the serial dilution method of TPL extract to determine MICs and MFCs_90_ for all fungal species and TPL extract dilution are presented in Table 2. Although six different incubation periods were assessed, the values presented were obtained after 24 h. In general, the results demonstrate a wide range of activity of the different polygodial concentrations and the species of fungi. The activity ranged from complete inhibition in *F. oxysporum* and *C. albicans* to no or low antifungal activity in *P. griseofulvum*.

**Figure 2:**
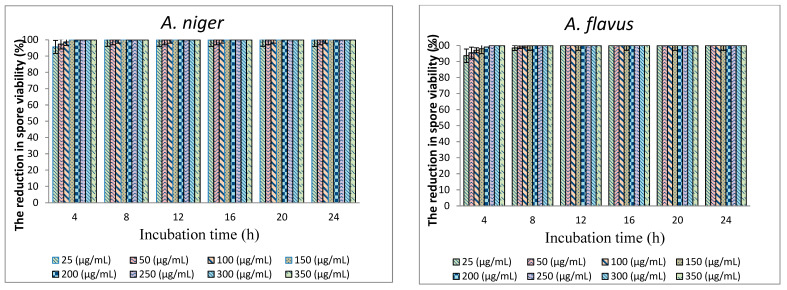

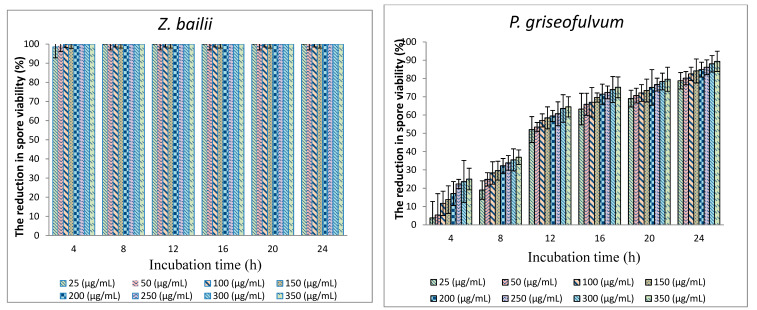

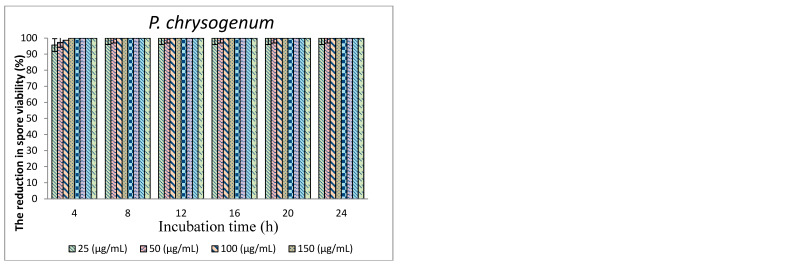
Effects of polygodial concentrations (25–350 µg/mL) found in TPL extract and incubation time on the spore viability of pathogenic and spoilage fungi.

The effects of polygodial concentrations in TPL extract and incubation periods on the spore viability of pathogenic and spoilage fungi are shown in Figure 2. The spores of *F. oxysporum* and *C. albicans* were very sensitive to all polygodial concentrations at all incubation periods with total inhibition (100%) (data not shown in graph). In contrast, the spores of *A. niger*, *P. chrysogenum,* and *Z. bailii* were inhibited at all polygodial concentrations when incubated for 8 h. At low concentration, this ranged from 25–100 µg/mL, and more than 93% reduction of spore viability of *A. niger*, *A. flavus*, *P. chrysogenum,* and *Z. bailii* was achieved after 4 h of incubation. 

A relatively high concentration of polygodial (i.e., 300 µg/mL) showed a reduction in *P. griseofulvum* spore viability of up to 89.38% after 24 h incubation. In contrast, the lowest concentration of polygodial (i.e., 25 µg/mL) could only reduce 3.77% of the spore viability under the same conditions. The finding obtained from the current study might be difficult to compare directly to the results of other studies because no literature has assessed the antimicrobial properties of Maltodextrin-TPL extract powder. However, considering the bioactive compound in TPL extract, polygodial has potent antifungal activity, whether extracted from TPL or other plant species. 

The MIC of all tested fungi incubated for 24 h with TPL extract was observed as <25 µg/mL polygodial. This result was in agreement with Yano, Taniguchi [15], who found the same value of polygodial as MIC toward *A.niger* and *P. chrysogenum*. In a previous report, the fungicidal activity of polygodial against *Saccharomyces cerevisiae* was described to be significantly affected by polygodial at a concentration of 3.13 µg/mL [20]. The MICs and MFCs_90_ of tested fungi were evaluated as less than 25 µg/mL polygodial for all species except *P. griseofulvum,* for which the MIC was >350, and the MFC_90_ was 350 µg/mL polygodial. Therefore, the concentration of polygodial with less than 25 µg/mL may possess further potency against a wide range of pathogenic and food spoilage fungi. Even though *P. griseofulvum* showed no inhibition at low concentration, the spore formation rate was obstructed or delayed after the treatment when compared to untreated fungi. 

In addition to fungal growth, sporulation of all tested fungi was also reduced by all given concentrations of TPL extract except *P. griseofulvum*. This indicates that TPL extract affected various stages of the development of microbiota associated with date fruits. A previous study that demonstrated the ability of polygodial against *C.albicans* was undertaken by Kubo and Himejima [21], who showed the MIC of *C.albicans* to be 3.13 µg/mL polygodial at 18 h incubation. This pathogen is widely used in the antimicrobial testing of plant extracts and other compounds because of its medical significance in causing opportunistic infections and food-borne diseases. Meanwhile, 50 µg/mL polygodial was reported as the MIC for *Z.bailii* [20]. Another study has reported the potency of polygodial against molds, including *P. chrysogenum* and *A. flavus,* which showed inhibition by moderate to low susceptibility to polygodial [11]. 

A constant polygodial concentration with the increase in incubation periods was accompanied by increases in microbial reduction. This may indicate that a more extended incubation period allows polygodial to disrupt the lipid-protein interface of integral proteins and denature their conformation [18,22]. Also, polygodial may enter through the cell membrane by passive diffusing and react with various intracellular components, affecting metabolic processes [23]. 

Another suggested target for polygodial could be the mitochondrial ATPase [24]. Several herbs, spices, and plants have been reported to be potential sources of antimicrobial agents, but not many have been studied with respect to levels and range of activity [25]. Overall, according to the result of both assays conducted in this study, it should be emphasized that the antifungal activity of TPL extract varied depending on polygodial concentrations in TPL extract, incubation periods, and fungal species.

### 3.2. Shelf Life Extension of Fresh Dates

There are different methods to preserve date fruits, such as refrigeration (by slowing down enzymatic reactions as well as the activity of microbes life), fumigation (killing insects at various stages of development), heat treatment (controls the Nitidulidae beetles by ensuring 100% mortality), modified atmosphere packaging (MAP) (to preserve the quality maintenance of dates with limited effects on the appearance of physiological disorder signs), edible coating (by improving the appearance of dates, protecting the fruits and reducing stickiness in case of soft dates), Ozone(O_3_) treatment (reduces or eliminates all life stages of Indian meal moth and sawtooth grain beetle), electron-Beams, the UV-C light (lethal to most of the microorganisms) and electrolyzed water [1].

**Figure 3:**
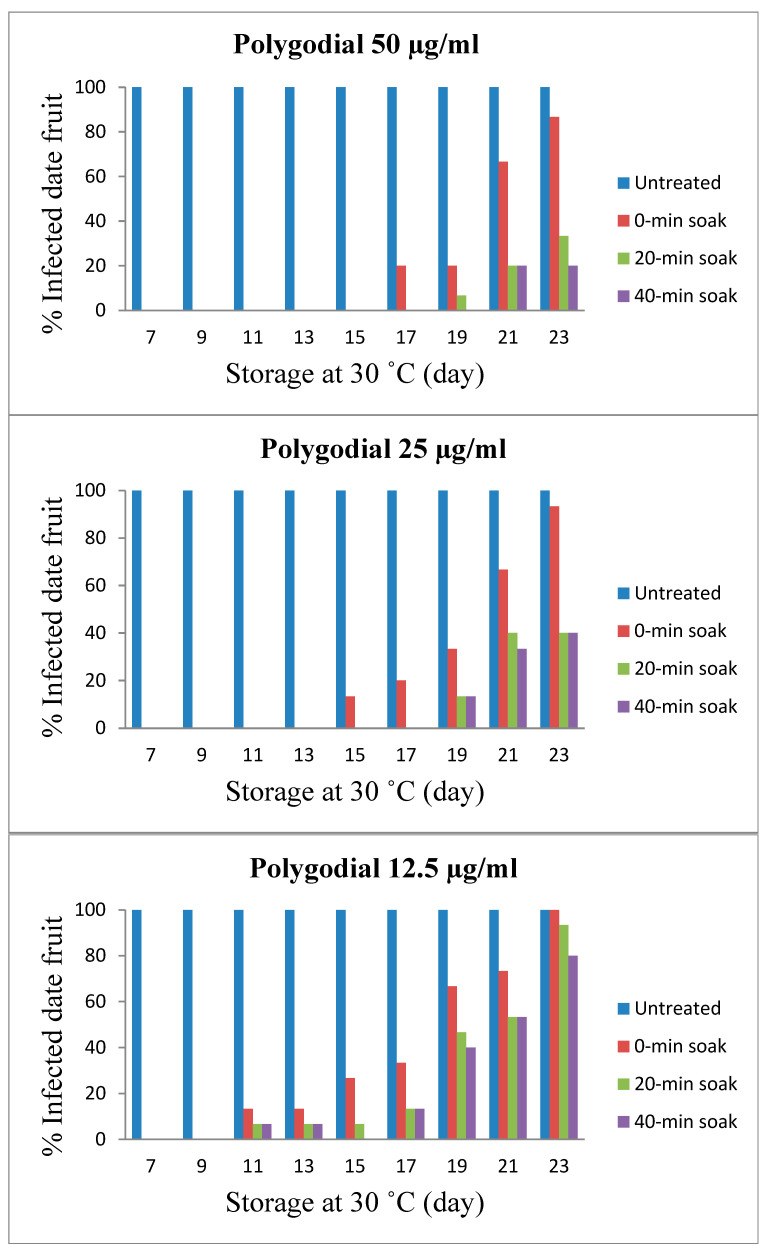
Effects of polygodial concentrations (12.5, 25, and 50 µg/mL) found in TPL extract extend the shelf life of fresh dates (*Barhi* cultivar) stored at 30 °C. Untreated dates lasted for 7 days before being infected by fungi. Increases in polygodial concentration and soaking period revealed increased shelf life of fresh dates.

Screening for antifungal activity of TPL extract at different concentrations of polygodial in extending the shelf life of fresh dates was assessed at 30 °C. The aim was to eliminate or reduce the mycobiota naturally contaminating the surface of fresh dates during 30 °C storage temperature, thereby extending the shelf life of fresh dates. The shelf life extension of date fruits is shown in Figure 3. In general, the shelf life of all treated fresh dates was extended up to double the time of control samples under the same conditions. Moreover, the effect of soaking time (contact time of the dates in various concentrations of TPL extracts) on the shelf life extension has also been studied. Soaking was performed for 0, 20, and 40 min. This study showed that the more the soaking was allowed, the more extended the shelf life. The dates treated with the highest concentration of TPL extract (corresponding to 50 µg/mL polygodial) followed by incubation at 30°C with no soaking lasted up to 17 days (20% of the fruit surface infected) compared to control samples that lasted for only 7 days (100% of the fruit surface infected) under the same conditions. Meanwhile, with soaking for 20 and 40 min, no fungal growth was observed for up to 19 days (7% infected) and 21 days (20% infected). The extent of fungal growth and no fungal growth were determined, followed by categories in Table 1, and the decision was made based on Table 1.

With a lower concentration of TPL extract (corresponding to 25 µg/mL polygodial), the shelf life of dates without soaking was 15 days (13% infected), whereas soaking for 20 and 40 min extended the shelf life by up to 19 days (13% infected) under the same conditions. The lowest given concentration of TPL extract (corresponding to 12.5 µg/mL polygodial) induced weak inhibition with only 11 days of shelf life for soaked and non-soaked fruit (7 and 13% infected, respectively) compared to 7 days of shelf life for control dates. TPL extract has been approved as a generally regarded safe (GRAS) flavoring ingredient by the Flavor and Extract Manufacturers Association (FEMA No. 4755) under the conditions of the intended use in food flavorings in accordance with the 1958 Food Additives Amendment to the Federal Food, Drug, and Cosmetic Act. 

Minimizing post-harvest losses caused by microbes of many agricultural crops is still a challenge. As inhibition of microbes in food may require high levels of plant extracts, interference with the flavor of the food product is likely. Thus, the level of plant extract typically added to foods is generally not enough to inhibit microorganisms completely but can inhibit spoilage to some extent [26]. Also, the efficiency and stability of plant extract may be influenced by the intrinsic (pH, salt, antioxidants, and other additives) and extrinsic (temperature, vacuum and modified atmosphere packaging, characteristics of microorganisms) properties of the food products [27]. Plant extract can be applied to the food product as natural preservatives or combined with a range of processes, including mild heat stress or MAP. 

The findings emphasized the time dependence of polygodial, in which more exposure to polygodial induces more microbial inhibition. Anke and Sterner [28] conclude that polygodial is highly effective as antimicrobial and cytotoxic properties of several similar compounds were found to have an antibiotic effect but no mutagenic capability. Utilizing TPL extract may help protect food commodities and fresh agricultural crops against the microbial activity, thereby extending shelf life and minimizing post-harvest losses. It should be noted that the naturally occurring mycobiota of fresh dates may also be seriously injured or stressed due to the frozen storage of dates before the experiment started and, therefore, may have been more sensitive to the TPL extract than inoculated pathogens.

## 4. Conclusions

The extract of TPL contained polygodial at various concentrations has the potential to control the growth of pathogenic and spoilage fungi, which could, in turn, lead to an extension of the shelf life of fresh dates. The viability of all tested fungi treated by TPL was inhibited or significantly reduced except *P. griseofulvum*. The shelf life of fresh dates treated by TPL extract (50 µg/mL polygodial) was extended up to 21 days at 30 °C storage temperature when dates were soaked for 40 min, and 19 days for dates soaked for 20 min, while the fruits which were not soaked were extended for 17 days compared to the 7 day shelf life of control fruits. 

Other concentrations of TPL extract (corresponding to 25 µg/mL polygodial) showed 15 days of shelf life with no soaking and 19 days for soaked fruits, while 12.5 µg/mL polygodial extended for only 11 days for soaked fruits and 7 days for non-soaked fruits under the same conditions. Overall, the shelf life of fresh dates treated by TPL extract at the concentration of 50 µg/mL polygodial at a storage temperature of 30 °C and soaking for 40 min was extended up to 21 days and was found to be the best results from the current study. The current work also clearly demonstrates that TPL extract containing polygodial has activity against a range of pathogenic and food spoilage fungi. Therefore, TPL extract can potentially extend shelf life or improve the safety of fresh dates. However, the wide variation in levels and range of activity also indicates that the application of the TPL extract will strongly depend on the specific fungal problem to be addressed.

Furthermore, the flavor effects of the extract on dates and the potential interaction of the antifungal compounds with components of food matrices, such as sugars, were not assessed in this study. These factors may strongly influence the applicability of the TPL extract in certain products and need to be investigated on a case-by-case basis.

## Figures and Tables

**Table 1:** Infected/non-infected dates by fungi show the guideline for determining the shelf life of dates after treatment.

Degree of Fungal Infection	Date Fruits			Consideration of Infection
No mycelia growth	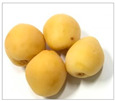	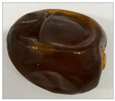	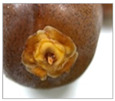	Not Infected 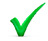
Slight mycelia growth	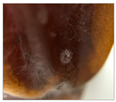	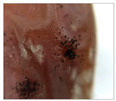	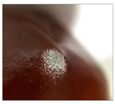	Infected 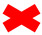
Moderate mycelia growth	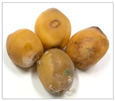	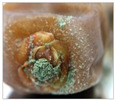	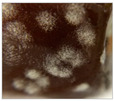	Infected 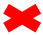
Severe mycelia growth	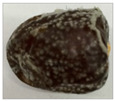	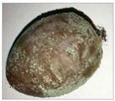	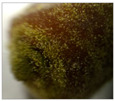	Infected 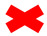

**Table 2:** Minimum inhibitory concentrations (MICs) and minimum fungicidal concentrations (MFCs) of polygodial found in TPL extract against pathogenic and spoilage fungi after 24 h incubation.

Polygodial (µg/mL)		
Fungi	MIC	MFC90
*A. niger*	<25	<25
*A. flavus*	<25	<25
*P. griseofulvum*	>350	350
*P. chrysogenum*	<25	<25
*F. oxysporum*	<25	<25
*C. albicans*	<25	<25
*Z. bailii*	<25	<25

MIC: when no visible growth is observed. MFC_90_: when 90% or more of fungi are inhibited.

## Data Availability

No new data were created or analyzed in this study. Data sharing is not applicable to this article.
